# Case of Cæsarean Section, in Which Both the Mother and Child Were Saved

**Published:** 1859-07

**Authors:** W. F. McClelland

**Affiliations:** Council Bluff, Iowa


ART. V.—*Case of Cæsarean Section, in which both the Mother and Child were saved.* By W. F. MCCLELLAND, M.D., of Council Bluff, Iowa.

ON the morning of the 9th of November, 1857, I was summoned to see Mrs. M., who had been taken in labor a few hours previously. The patient was about thirty years of age, had arrived at her full time, and presented all the symptoms of a speedy delivery. Upon attempting, however, to make an examination per vaginam, I found, about an inch from the vulva, an obstruction which would not allow the passage of the finger. The obstacle felt very much like a thickened, unbroken hymen, with an opening in the centre, through which I could barely insinuate the point of my finger. Upon questioning the patient, I learned that she had been confined but once before, about five years previous, at which time the physicians in attendance were obliged to remove the child "in pieces," and that the instruments employed for the purpose produced great laceration of the vagina.

Judging from the history thus briefly related, that there might be deformity of the pelvis, as well as stricture of the vagina, I called in consultation my partner, Dr. Horne, who advised that the labor should be allowed to progress until the head distended the vagina, when the stricture should be divided. To this I readily consented, and permitted the case to continue for several hours, at the end of which time, although the pains had been strong, I found, upon examination, that the head had not descended into the pelvis. I then determined to divide the adhesions of the vagina, in order to permit an examination beyond. This was accomplished by means of a probe-pointed bistoury, wrapped to within half an inch of the point, and the constriction thus incised at two points. Upon introducing the finger, I could now feel the mouth of the uterus, found it dilatable, and the vertex presenting, but unable to descend in consequence of great deformity of the pelvis. As nearly as I could ascertain, the antero-posterior diameter of the pelvic canal did not exceed one and five-eighths inch, and the transverse one and a half inch. The sacrum also was very much curved, and the angle of the pubic arch very acute. The uterine pains had now been strong for fifteen hours, when I again called Dr. Horne in consultation. After a renewed examination of the parts, we concluded that the pelvic canal was not only too small to allow the passage of the child in the usual way, but also too narrow to justify a resort to the perforator and crotchet. The only alternative was the Cæsarean section, and having decided upon this, I reported the fact to the patient, who consented, without a moment's delay, to submit to our decision.

The bladder having been evacuated, and the other usual preliminaries attended to, the patient was placed upon her back, the shoulders slightly raised, and chloroform administered by Dr. Grimes, who was invited to be present. I then made an incision along the linea alba, extending from about an inch below the umbilicus to within two inches of the symphysis pubis, dividing the whole thickness of the abdominal parietes. A corresponding incision of the uterus was next made, measuring about six inches in length, in performing which I first made a small opening above and continued it downward, using the index finger of my left hand as a director. The child was easily extracted, and cried lustily, and while I secured the cord, Dr. Horne removed the placenta. As soon as the latter was accomplished, a tolerably profuse hemorrhage took place, but by the application of sponges wrung out of cold water, the uterus was soon made to contract, and all bleeding ceased. Having carefully put back the omentum out of the way, I brought the external wound together by interrupted sutures and adhesive strips, leaving the lower angle open. A full anodyne was next administered, and during the succeeding night the patient rested as well as could have been expected.

The following morning she appeared quite comfortable; pulse 96.

Second day, pulse 106; considerable fever; great tenderness of the abdomen, and tympanites. Ordered Dover's powder, grs. xii, calomel, grs. xx, with fomentations to the abdomen. A few hours after, the bowels moved three times; fever subsided; pulse reduced to 100; tympanites began to disappear. The succeeding night she slept well. The lochia passed entirely by the vagina.

The third day after the operation milk appeared in the breasts, and the child was nursed.

From that time no unpleasant symptom occurred, and by the end of the second week the wound was entirely closed. The third week the patient sat up in bed, and in less than ten days thereafter she was about the house attending to her domestic duties.

She has now, December sixteenth, entirely recovered; and the child is growing finely.

